# Stochastic Evolution Dynamic of the Rock–Scissors–Paper Game Based on a Quasi Birth and Death Process

**DOI:** 10.1038/srep28585

**Published:** 2016-06-27

**Authors:** Qian Yu, Debin Fang, Xiaoling Zhang, Chen Jin, Qiyu Ren

**Affiliations:** 1 School of Economics, Wuhan University of Technology, Wuhan, 430070, China; 2 School of Economics and Management, Wuhan University, Wuhan, 430072, China; 3 Department of Public Policy, City University of Hong Kong, Hong Kong, China; 4 Department of Economics, The George Washington University, Washington DC, 20052, United States

## Abstract

Stochasticity plays an important role in the evolutionary dynamic of cyclic dominance within a finite population. To investigate the stochastic evolution process of the behaviour of bounded rational individuals, we model the Rock-Scissors-Paper (RSP) game as a finite, state dependent Quasi Birth and Death (QBD) process. We assume that bounded rational players can adjust their strategies by imitating the successful strategy according to the payoffs of the last round of the game, and then analyse the limiting distribution of the QBD process for the game stochastic evolutionary dynamic. The numerical experiments results are exhibited as pseudo colour ternary heat maps. Comparisons of these diagrams shows that the convergence property of long run equilibrium of the RSP game in populations depends on population size and the parameter of the payoff matrix and noise factor. The long run equilibrium is asymptotically stable, neutrally stable and unstable respectively according to the normalised parameters in the payoff matrix. Moreover, the results show that the distribution probability becomes more concentrated with a larger population size. This indicates that increasing the population size also increases the convergence speed of the stochastic evolution process while simultaneously reducing the influence of the noise factor.

Cyclic competition is a very common phenomenon in nature and society, with the simplest and most studied model being the Rock-Scissors-Paper (RSP) game^1^. This can be characterised by three strategies: R (rock), S (scissors) and P (paper), where R excludes S, S excludes P and P excludes R. Because of their widespread existence, RSP games have attracted the attention of many researchers in related fields – being widely applied in biological systems, such as biological diversity^2^,^3^ and species interaction^4^.

Schreiber and Killingback^5^, for example, have studied the effect of a general metacommunity structure on the coexistence of the strategies in the RSP game to show that dispersal in spatially heterogeneous environments can alter dynamical outcomes; Jeppe^6^ quantitatively describes how spatial clustering slows down the dynamics and stabilizes the spatial system of the game; and Kerr and Riley^7^ and Kirkup and Riley^8^ show that three strands of E. colibacteria compete in a RSP fashion to reach an evolutionary stable distribution. The RSP game can also be applied to economic and social systems as well as biological systems. In the Edgeworth price cycle, for example, when not all consumers are fully informed with pricing information, oligopolistic pricing can be regarded as a RSP game^9^. Also, the RSP cycle arises in public goods games^10^,^11^, where the populations are separated into three groups: co-operators, defectors and loners.

Various evolution properties of population behaviour for the RSP game have been analysed based on evolutionary dynamics such as the replicator dynamic and the Smith dynamic, the BNN dynamic and the BR dynamic, etc. after evolutionary game theory was developed. Around the 1990 s, Hofbauer^12^ studied the evolutionary stability of the Nash equilibrium under the replicate dynamic and proved that the Nash equilibrium of the RSP game is just the fixed point of the replicate dynamic. Meanwhile, Weibull’s^13^ analysis showed that any evolutionary stable profile of the RSP game is not only a fixed point under the replicator dynamic but is also asymptotically stable. Furthermore, Gaunersdorfer and Hofbauer^14^ found that the limiting behaviour of fictitious play and the time-averaged replicator dynamics coincide in the RSP game, while Loertscher^15^ proved it has an evolutionary stable strategy (ESS) when the discount factor of the game is less than 1.

However, deterministic models can no longer be relied upon for finite populations. In this case, the random fluctuations due to sampling effects, for instance, have to be taken into account and stochastic processes, such as the Moran process and the Wright-Fisher process, must be employed to analyse the equilibrium of RSP^16^,^17^ instead of ordinary differential equations.

Studies of the impact of stochasticity on the RSP game indicate various important factors influencing the cyclic behaviour of populations, such as noise, alliance-specific heterogeneous invasion rate, mutations, group interaction and protection spillovers except evolutionary dynamics. Perc and Szolnoki^18^,^19^, for example, have studied noise-guided evolution in a six-species predator-prey model within cyclical interaction and cyclical interaction with alliance-specific heterogeneous invasion rates; Mobilia^20^ studied the oscillatory dynamics in generic three-species RSP games with mutations; and Szolnoki^21^,^22^ considers group interaction in RSP games and studied the vortices to determine the dynamics of biodiversity in cyclical interactions with protection spillovers. Szabó and Fáth^23^ discuss the evolution dynamic of the RSP game in a lattice grid and depict heat maps of the evolutionary dynamic based on the Monte-Carlo simulation method. The limit cycles have also been found for the RSP game by Ochea^24^ under logit dynamics.

Although these studies have been very successful and well verified in the evolutionary biology field, the RSP game played by the individuals with bounded rationality in a social and economic system still needs to be investigated, because the learning dynamic in such situations may differ from that based on genetics. Motivated by this question, research attention turned to focus on the evolutionary RSP game under learning dynamics, such as imitation^25^ and best-response^26^. Sato *et al*.^27^, for instance, investigated the RSP game based on the reinforcement learning dynamic, finding the learning trajectory can be simple or complex depending on initial conditions, and that the learning process displays Hamiltonian chaos for the zero sum game; while Platkowski and Zakrzewski^28^ found an asymptotically stable polymorphism for the standard RSP game and the limit cycles for the general RSP game under complex personality profiles of the players and the imitation dynamics. In 2014, Szolnoki^21^ reviewed the main results in well-mixed populations with the focus on oscillatory dynamics and firstly on the important role of the topology of the interaction networks^29^. Hoffman *et al*.^30^ experimentally investigated three RSP games and found population distributions further from the centre are more frequent when the NE section is not stable, which is consistent with the predictions of non-deterministic evolutionary dynamics models.

To analyse an evolutionary model with a finite number of players and with noise or mutations, Kandori^31^ firstly considered stochastic characteristics in the learning dynamics and introduced the Markov process model to describe the learning dynamic in evolutionary games. Amir and Berninghaus^32^ extended this to a continuous time model^33^ and modelled the evolutionary process of the evolutionary normal-form game with a finite number of players as a homogeneous Markov process with finite state space. Tadj and Touzene^34^ then proposed the state dependent quasi-birth-and-death process model to describe the learning dynamic for more complicated stochastic evolution. Compared with general 3^*^3 game described in Tadj and Touzene^34^ that emphasises computational efficiency, this paper is more focused on the impact of stochasticity on the evolutionary dynamic of the RSP game, which considers population size, the payoff matrix and noise factor. In addition, according to the unique characteristics of the RSP game, we consider that the states above the diagonal can be transferred to each other.

To study the stochastic evolutionary dynamic of the RSP game in finite populations and analyse the effect factors of the stochastic evolutionary dynamic, we assume that the bounded rational individuals in a finite population can adjust their strategies by imitating the successful strategy according to payoffs from the last round of the game, and model the stochastic evolutionary process involved as a finite, state dependent, quasi birth and death process. The long run equilibrium of the RSP game played by bounded individuals in a finite population can be explained by the limiting distribution of the QBD process of the game evolutionary dynamic. With the limiting distribution, the probability of a stable state of the RSP game evolution dynamic can be more profoundly predicted and interpreted. Furthermore, comparisons of the numerical experimental results plotted as pseudo colour ternary heat maps shows the effect of the population size and parameters in the RSP payoff matrix and noise factor.

## Results

### Nash equilibrium of the general RSP game

A symmetrical Rock-Scissors-Paper game can be characterised by three strategies R, S and P. Each strategy dominates the next one in a cyclical way.

The standard RSP game can be depicted by the constant-sum payoff matrix


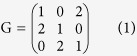


It is easily verified that this game has no Nash equilibrium in pure strategies and precisely one Nash equilibrium in mixed strategies, namely the strategy pair in which both players randomise uniformly, i.e., 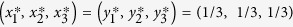.

Furthermore, the payoff matrix of a general Rock-Scissors-Paper game can be normalised as:


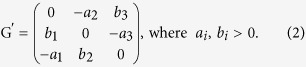


It is easy to see that there always exists a unique Nash equilibrium that is


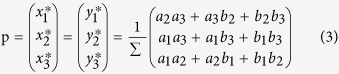


where Σ is a normalising constant.

Under the replicator dynamic, the unique Nash equilibrium p, is asymptotically stable when 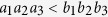, the Nash equilibrium is neutrally stable when 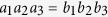 and the Nash equilibrium is unstable when 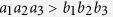
35.

With a special transformation, we can obtain a simple payoff matrix for the general RSP game G′ as:


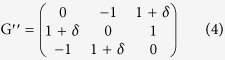


**Figure 1:**
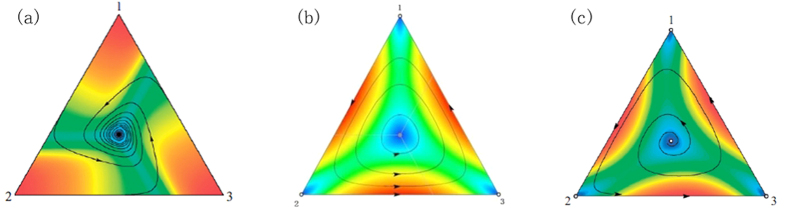
Phase portraits of the RSP game in replicator dynamics^24^. In replicator dynamics, the Nash equilibrium (**a**) will be asymptotically stable and the dynamical trajectory will converge to the centre of the simplex if and only if δ > 0. While as (**b**), the dynamical trajectory will converge to a periodic orbit and obtain the state of neutrally stable if δ = 0. For (**c**), the trajectory will diverge from the centre point and converge to the boundary of the simplex if δ < 0.

Then, the Nash equilibrium becomes 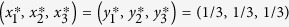. Coding with Matlab 7, the phase portraits of the RSP game are shown in Fig. 1.

### Numerical experiments for the symmetrical RSP game

We can model the stochastic evolutionary dynamic of the RSP game as a QBD process and carry out numerical simulations to verify the dynamic stability of long run equilibrium for the RSP game in populations. The limiting distributions of the QBD process can be solved for the RSP game stochastic evolutionary dynamic (see Methods section below).

**Figure 2:**
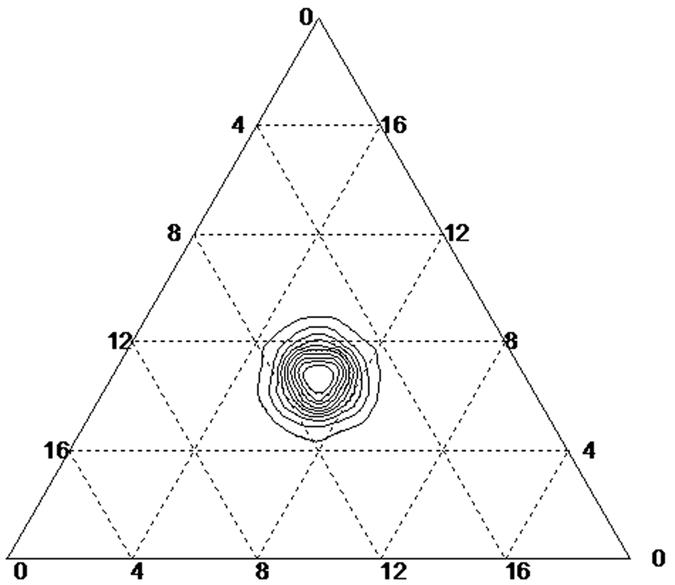
Contour diagram of the limiting distribution of the QBD process. The contour diagram of the QBD process causes the limiting distribution of the general RSP game *G*″ when N = 20, δ = 5 and ε = 0.01, where the contour lines mean the states have the same probability in the limiting distributions. The diagram shows that the states in the centre of the simplex have the highest probabilities.

For example, by setting N = 20, δ = 5 and ε = 0.01, the limiting distributions of the QBD process of the general RSP game *G*″ can be obtained. Then the profiles, whose appearance probability is larger than 0.01, are listed in Table 1. For instance, there are 7, 6 and 7 individuals in the population in row 1 who chose strategy R, S and P respectively and 0.1461 is the appearance probability of this state in the QBD process. As shown in Fig. 2, the main probability in the limiting distribution of the QBD process is concentrated in the states around (7,6,7), (7,7,6) and (6,7,7). The sum of these probabilities is 0.8876, indicating that (1/3, 1/3, 1/3) can be regarded as the long run equilibrium of the stochastic evolutionary RSP game.

**Figure 3:**
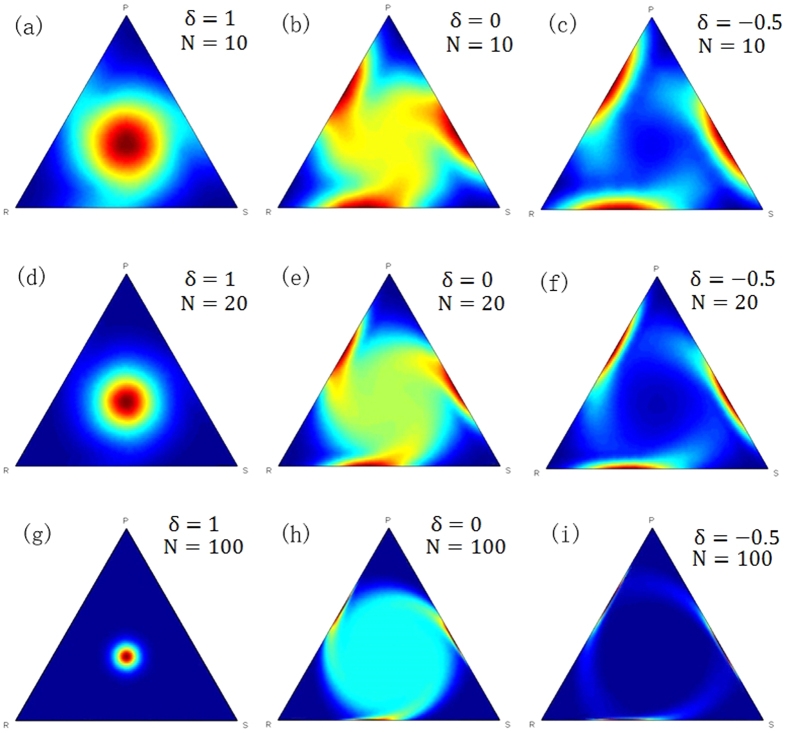
Pseudo colour ternary heat maps of simulations for ***G***′″. The numerical experiments based on the QBD process were carried out for simulating the stochastic evolutionary dynamic of RSP game, with the different population size of 10, 20, 100, and different payoff parameter of −0.5, 0, 1, respectively. In these pseudo color ternary heat maps, each point in the simplex corresponds to mixed strategies of RSP game. The hue value of each point, ranging from red to blue, indicates the probability of the corresponding strategy in the limiting distribution of QBD process. By comparing (3a) to (3i), we can see (A): The heat map appeared as a target when δ > 0 (3a, 3d, 3g). In the diagram, the red points gathered around the center indicate that the point (1/3, 1/3, 1/3) has the highest probability in the limiting distribution which confirm the Nash equilibrium is an asymptotically stable equilibrium. The heat map appeared as a nebula with three spiral arms when δ = 0 (3b, 3e, 3h), which corroborate that the center is merely neutrally stable. Moreover, the spiral arms far from the center when δ < 0 (3c, 3f, 3i) demonstrates that the long run equilibrium will be a mixed-strategy equilibrium on the boundary of the simplex. (B): The states corresponding to the points have more concentrated distribution probability when the size of the population is100 rather than 20 and 10, which indicates that the larger the size of the population, the convergence speed of the stochastic evolution process will be faster and the influence of perturbation will be weaker.

To analyse the results more intuitively and compare the effect of population size and the payoff on the QBD process, we carried out numerical experiments with sizes 10, 20 and 100, and set the payoff parameter δ as −0.5, 0 and 1 respectively. The noise factor was set at 0.01. The limiting distribution of QBD was then obtained and plotted as pseudo colour ternary heat maps (Fig. 3), where the vertical distances of each point to the three edges in the simplex represents the probability of choosing either the R, S or P strategy respectively. Therefore, the top, bottom left and bottom right vertices of the simplex correspond to the three pure strategies (1, 0, 0), (0, 1, 0) and (0, 0, 1) respectively, and other interior points correspond to other mixed strategies. Figure 3 shows the results, where the hue value of each point, ranging from red to blue, indicates the probability of the corresponding strategy in the limiting distribution of the QBD process.

Comparison of these results indicates that:

1. Observing Fig. (3a,3d,3g), we can see when δ = 1 or δ > 0, the heat maps appear as a target, the red points representing the states with higher distribution probability gathered around the centre of this simplex regardless of population size. Most of the remaining points appear as blue, which means the distribution probabilities of the states corresponding to those points approach zero. These features confirm that the mixed strategy profile (1/3, 1/3, 1/3) is a long run Nash equilibrium of the RSP game and also an asymptotically stable equilibrium when δ > 0.
2. Observing Fig. (3b,3e,3h), the colour images appear as a nebula with three spiral arms, with the colour of the unscrewing arms from the centre to the edges of the simplex gradually turning red. The impression is that the interior points in the simplex are simultaneously attracted by forces from the centre point and centrifugal force from the edges of simplex. The change of colour shows that the distribution probabilities of states corresponding to the points are increasing along the spiral arms of the nebula. This corroborates that, when δ = 0, the Nash equilibrium (1/3, 1/3, 1/3) is merely neutrally stable, and the states corresponding to the points on the central sections of the edges occur with higher distribution probability.
3. Observing Fig. (3c,f,i) when δ = −0.5 or δ < 0, the red points representing those states with higher distribution probability are gathered around the area near the centre sections of the edges in the simplex. The rest of the parts in the simplex are almost blue, which means the states corresponding to these points have distribution probabilities approaching zero. This indicates an unstable evolutionary Nash equilibrium with condition δ < 0. The long run equilibrium will be a mixed-strategy on the boundary of simplex, such as (2/5, 3/5, 0), (0, 2/5, 3/5) and (3/5, 0, 2/5) in the simulation experiment.
4. Vertically comparing diagrams from (3a) to (3g), (3b) to (3h) and (3c) to (3i), indicates larger populations have more concentrated distribution probabilities. This indicates, along with increasing population size, the convergence speed of stochastic evolution process will be faster and the influence of the perturbations will be weaker.

### Numerical experiments for the asymmetrical RSP game

To avoid artificial symmetries structure, we also investigated the asymmetrical RSP game as^36^:


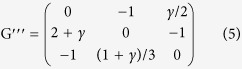


Under the replicator dynamic, G′″ has a saddle node fixed point at the vertices of the simplex and an interior fixed point at (1/2, 1/3, 1/6), independent of γ. However, the stability of the fixed point will be affected by γ. For γ > 1, (1/2, 1/3, 1/6) is a stable focus, and an unstable focus for γ < 1. The figure will exhibit closed orbits when γ = 1.

**Figure 4:**
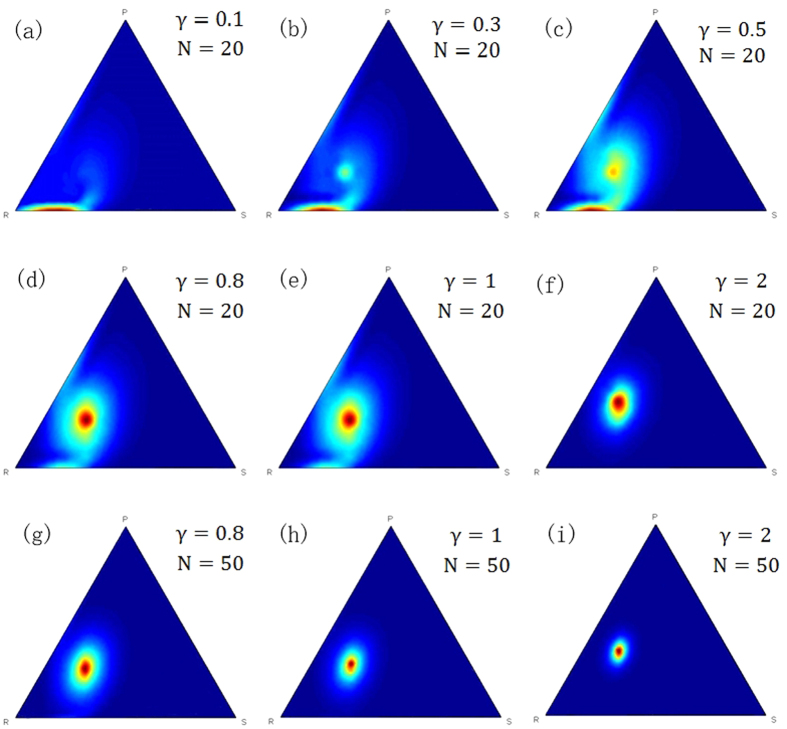
Pseudo colour ternary heat maps of simulations for *G*′″. The asymmetrical RSP game was played in the population with sizes of 20 and 50. The payoff parameters were set as 0.1, 0.3, 0.5, 0.8, 1 and 2 respectively. Fig. (4a–f) show the red points gathered together at the left bottom edge in the simplex at the beginning, and the vortex appear gradually and screwed to the point near (1/2,1/3,1/6) with the increasing of *γ*. Under the QBD process evolution dynamic, the long run equilibrium will be a mixed-strategy equilibrium on the boundary near the left bottom corner in the simplex when *γ* is small. The Nash equilibrium will became the long run equilibrium eventually if *γ* ≥ 1. Figure 4d–i show that the larger the size of the population, the stronger the vortex will be. This indicates, along with the increasing population size, the convergence speed of stochastic evolutionary process will be faster and the influence of noise will be weaker.

Based on the QBD model of RSP game, we set the population to 20 and 50, and the parameter γ in the payoff matrix as 0.1, 0.3, 0.5, 0.8, 1 and 2 to inspect the effect of γ. The perturbation factor was set to ε = 0.01 to represent noise. The resulting pseudo colour ternary heat maps are shown in Fig. 4.

From these, we find that:

(1) Observing (4a–f), when γ < 1, the red points representing the states with higher distribution probability are gathered at the left bottom edge in the simplex at the beginning, and the vortex appears gradually and screwed to the point near (1/2,1/3,1/6) with increasing γ. The rest of the parts in the simplex are almost blue, indicating the states corresponding to the points have distribution probabilities approaching zero. Therefore, the long run equilibrium will be a mixed-strategy equilibrium on the boundary near the left bottom corner in the simplex when γ is small and the Nash equilibrium will become the long run equilibrium eventually when γ ≥ 1. The Nash equilibrium at (1/2,1/3,1/6) with the condition γ < 1 is an unstable evolutionary equilibrium, will become neutrally stable when γ = 1 and will be an asymptotically stable equilibrium finally when γ > 1.

(2) Vertically comparing from (4d) to (4i), increasing the population size strengthens the vortex. This indicates that, as with the symmetric situation in Fig. 3, as the population size increases, the convergence speed of stochastic evolutionary process is faster and the influence of noise is weaker.

## Discussion

In this paper, we established a QBD process for the stochastic evolutionary dynamic for the RSP game. For the general RSP game *G*″, the pseudo colour ternary heat maps of numerical experiments show that the limiting distribution will converge to the centre of the simplex when δ > 0, diverge as spiral nebula when δ = 0 and diverge to the boundary of the simplex when δ < 0. For the asymmetrical RSP game *G*′″, the limiting distribution will converge to the Nash equilibrium (1/2, 1/3, 1/6) gradually when γ > 1, become neutrally stable when γ = 1 and diverge to the boundary of the simplex when γ < 1. The numerical experimental results are consistent with the conclusions proposed in the literature^12^,^13^ based on replicator dynamics.

Moreover, the study provides a deeper understanding of the RSP game in three aspects. Firstly, the stochastic evolutionary dynamic of the RSP game here was built based on the assumption of bounded rationality. This assumption makes the model more suitable for describing an individual’s behaviour in social environments and gives the study more practical significance. In fact, as Zhijian Wang^37^ recently revealed in a laboratory experiment of the discrete-time iterated RSP game, a microscopic model of a win-lose-tie conditional response can offer higher payoffs to individual players in comparison with the NE mixed strategy, suggesting that high social efficiency is achievable through an optimised conditional response. Based on the assumption of bounded rationality, the numerical experiment results in Fig. 3 are very similar to the laboratory experimental evidence observed by Moshe^30^.

Secondly, the probability interpretation for the long run equilibrium of the RSP game can be provided by the limiting distribution of the QBD process. The limiting probabilities of the states in the QBD process can be used to predict how likely the equilibrium of the RSP game will be.

Finally, the noise factor introduced in our model can be used for detecting the stochastic stability of the long run equilibrium in evolutionary dynamics, which could not be done by previous research with replicator dynamics.

In summary, our study provides a new means of interpretation for the long-term equilibrium of the RSP game played by bounded rational individuals, based on learning dynamics. Further research is needed to generalise these results. For example, RSP games that take place between players from heterogeneous populations can be taken into account. The learning dynamic contained in the QBD process of our model can also be further generalised.

## Methods

Without loss of generality, we supposed that the general RSP games are repeatedly played between individuals in a finite population with a size of *N* and payoff matrix *G*′.

First, denote the three strategies R, S and P in the RSP game as 1, 2 and 3. Then we can define a stochastic process as: the state at time, t, is a two-dimensional stochastic variable 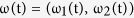, where 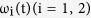 are the number of players in the population who choose strategies R and S, while the number of players in the population who choose strategy P is 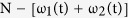. Hence, the profile and the average payoffs of players are decided by 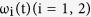 and 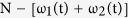 at time t.

At time t, if there are 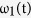 individuals choosing strategy R, 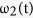 individuals choosing strategy S and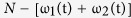 individuals choosing strategy P, then any individual in the population adopting strategy i will obtain an average payoff 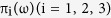, where 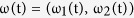:

if the individual chooses strategy R,


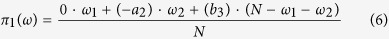


if the individual chooses strategy S,


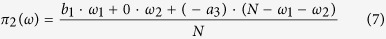


and if the individual chooses strategy P,


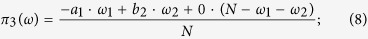


The next time, *t* + *1*, the individuals in the population will switch from one strategy to another at a rate depending on the average payoff of each strategy at time *t*.

According to the three hypotheses of bounded rationality proposed by Kandori^31^, the individuals in the population are inertia, myopia and mutation. That means that not all players react instantaneously to their environment; the players are not taking into account the long run implication of their strategy choices; and the players will change their strategies at random with a small probability.

According to the profile and the average payoffs at time *t* we can suppose only one individual can change his strategy within a short time. That is, at time *t* + *1*, 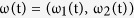 can only shift to an adjacent state. In this case, the strategy distribution of the population satisfies the inertia hypothesis. The myopia hypothesis will also be satisfied because only the profile and the average payoffs at time *t* will be considered by the individuals when they make the choice at time *t* + *1*. Furthermore, the mutation feature is introduced into the model by a perturbation factor ε ≥ 0, which allows the players to deviate from the selected strategy with a small probability.

Assume 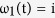 and 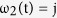, then the individuals in the population will switch from strategy 1 to strategy 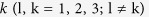
at the rate 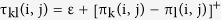, where 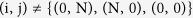, and 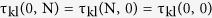, where 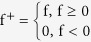 is defined for an arbitrary real function f.

This means the individuals choosing strategy 1 will switch to strategy k at rate 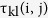, if 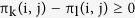, and the transition rate will be 0, if the number of individuals adopting the same strategy in the population reach N, the size of the population.

The stochastic evolutionary process of the strategy distribution of the RSP game then becomes a two-dimension Markov stochastic process that can be described as a QBD process.

Denote the state space of 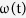 as 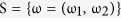, where 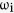 denotes the number of individuals choosing strategy i in the population (i.e. strategy distribution at time t). Obviously the number of states is 
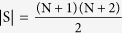
. Given 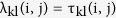, k < 1 and 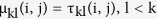, the evolutionary process of strategy distribution is a finite, state dependent QBD process, better known as a generalised QBD process (GQBD).

**Figure 5:**
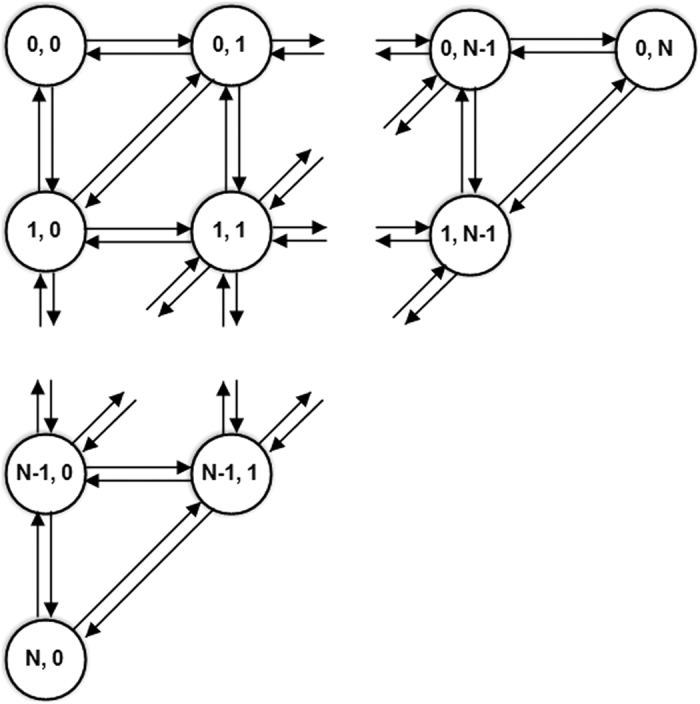
Switches between states in the GQBD.

The switches between states in this GQBD can be as depicted in Fig. 5.

The Q-matrix of the GQBD is then:


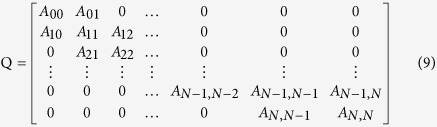


Using a numerical algorithm based on the block Gauss-Seidel iteration method, proposed by Stewart^38^, the steady-state probability of the GQBD can be obtained if the GQBD is recurrent. The solution is the steady distribution of the GQBD in the long run, which is a limiting distribution of strategies of the RSP game. In fact, it can be considered as the long run equilibrium of the stochastic evolution RSP game in the population.

## Additional Information

**How to cite this article**: Yu, Q. *et al*. Stochastic Evolution Dynamic of the Rock–Scissors–Paper Game Based on a Quasi Birth and Death Process. *Sci. Rep.* **6**, 28585; doi: 10.1038/srep28585 (2016).

## Figures and Tables

**Table 1:** Main probabilities in the limiting distribution.

R	S	P	Limiting probability
7	6	7	0.1461
7	7	6	0.1461
6	7	7	0.1461
6	6	8	0.0646
8	6	6	0.0646
6	8	6	0.0646
7	5	8	0.0325
8	7	5	0.0325
5	8	7	0.0325
8	5	7	0.0287
5	7	8	0.0287
7	8	5	0.0287
5	6	9	0.0122
6	9	5	0.0122
9	5	6	0.0122
6	5	9	0.0117
5	9	6	0.0117
9	6	5	0.0117
…	…	…	….
